# Cross-continental immunity: unraveling hematological markers of HIV in Africa and Russia- a perspective

**DOI:** 10.1097/MS9.0000000000003617

**Published:** 2025-07-18

**Authors:** Emmanuel Ifeanyi Obeagu

**Affiliations:** Department of Biomedical and Laboratory Science, Africa University, Mutare, Zimbabwe

**Keywords:** African populations, hematological markers, HIV, immunity, Russian populations

## Abstract

Hematological abnormalities are common in individuals living with HIV, and these complications vary significantly across global populations due to diverse genetic, environmental, and socio-economic factors. This review explores the divergent immunohematological trends observed in HIV-infected populations in Africa and Russia, focusing on key hematological markers such as anemia, thrombocytopenia, and neutropenia. While both regions experience high burdens of HIV, their populations present with distinct hematological manifestations influenced by co-infections, lifestyle factors, and local healthcare systems. In Africa, HIV-associated hematological abnormalities are often compounded by endemic co-infections such as malaria and tuberculosis, which exacerbate anemia and thrombocytopenia. Additionally, poor nutritional status in many sub-Saharan African countries contributes to the severity of these complications. Conversely, in Russia, hematological complications in HIV-positive individuals are frequently linked to alcohol use and liver disease, with these factors playing a significant role in the pathogenesis of anemia and thrombocytopenia. The combination of HIV, alcohol-related comorbidities, and intravenous drug use complicates the immunohematological landscape in this region, requiring a more multifaceted approach to treatment.

## Introduction

HIV (human immunodeficiency virus) remains one of the most significant global health challenges, affecting millions worldwide. While the virus does not discriminate in its capacity to infect individuals, the way it impacts immune function and the hematological system varies dramatically across populations. These variations are influenced by a combination of genetic predispositions, environmental factors, socio-economic conditions, and the presence of co-infections or comorbidities^[1–3]^. Hematological abnormalities, such as anemia, neutropenia, and thrombocytopenia, are common among individuals living with HIV, yet the prevalence, severity, and underlying causes of these conditions differ across regions. This review aims to explore these divergent immunohematological trends by examining HIV-infected populations in Africa and Russia, regions that, despite both facing significant HIV epidemics, present distinct immunological profiles^[4,5]^. Africa is home to the majority of the global HIV burden, with sub-Saharan Africa accounting for the highest proportion of HIV-infected individuals. The region’s HIV epidemic is characterized by a unique combination of genetic diversity, high rates of co-infection with diseases such as tuberculosis and malaria, and socio-economic factors that influence healthcare access. These factors exacerbate the hematological complications of HIV, contributing to the increased prevalence of anemia, thrombocytopenia, and neutropenia. The interplay between HIV and endemic co-infections complicates the clinical picture, necessitating a multi-faceted approach to treatment that addresses both the HIV virus and these additional health burdens^[6,7]^.

In contrast, Russia’s HIV epidemic has its own distinct characteristics. Although the country has seen a rise in HIV prevalence, the primary risk factors for HIV in Russia are associated with intravenous drug use, alcohol abuse, and the social stigma surrounding the virus. These factors have contributed to an HIV epidemic that is deeply intertwined with alcohol-related comorbidities, including liver disease, which significantly affects hematological health. The higher rates of alcohol consumption and intravenous drug use in Russia complicate the diagnosis and management of HIV, making it necessary to consider alcohol-related liver damage and drug-induced immunosuppression when assessing hematological markers. Additionally, the healthcare infrastructure in Russia faces challenges in addressing these intertwined health issues, further exacerbating the complexity of managing HIV in this population^[8–11]^. While HIV’s effects on the immune system are well documented, the hematological implications are less frequently emphasized despite their significance. HIV infection can lead to bone marrow suppression, impacting the production of red blood cells, white blood cells, and platelets. This can result in a range of hematological disorders, including anemia (low red blood cell count), neutropenia (low white blood cell count), and thrombocytopenia (low platelet count). These conditions can significantly impact the individual’s ability to fight infections, increasing susceptibility to both opportunistic infections and bleeding complications. However, in regions like Africa and Russia, where the disease burden and social determinants of health vary significantly, the pathogenesis of these conditions and their management strategies also differ^[12–14]^.

In African populations, the burden of co-infections such as tuberculosis, malaria, and helminthic infections further complicates the hematological impact of HIV. For example, malaria-induced hemolysis and inflammation can aggravate anemia in individuals already infected with HIV, while tuberculosis, a leading cause of morbidity and mortality in HIV-infected individuals, can result in severe immunosuppression, leading to more pronounced hematological abnormalities. Additionally, nutritional deficiencies, including iron, folate, and vitamin B12 deficiencies, are common in many African countries and can worsen the severity of HIV-related anemia. These co-infections and deficiencies must be considered when diagnosing and managing HIV-related hematological abnormalities in African populations^[15–17]^. Russia’s HIV epidemic, on the other hand, is marked by a different set of challenges. The high rate of intravenous drug use in the country has resulted in a growing number of individuals co-infected with HIV and hepatitis C, further complicating the hematological landscape. Chronic alcohol use is also a significant factor in Russia, with alcohol-induced liver disease contributing to anemia and thrombocytopenia, common hematological manifestations in HIV-infected individuals. In these cases, the liver’s impaired ability to produce thrombopoietin, a hormone critical to platelet production, results in thrombocytopenia. Moreover, alcohol’s toxic effect on the bone marrow can directly suppress blood cell production, compounding the hematological complications associated with HIV^[18–20]^. The disparities in hematological manifestations between African and Russian populations underscore the need for region-specific diagnostic and therapeutic strategies. In Africa, the management of HIV requires not only antiretroviral therapy (ART) but also a comprehensive approach that addresses co-infections such as tuberculosis and malaria. This requires an integrated care model that combines ART with treatments for these co-infections. In contrast, Russian healthcare providers must account for the complex interactions between HIV, alcohol-related liver disease, and intravenous drug use. Here, the treatment approach must address not only HIV but also alcohol consumption, hepatitis co-infections, and liver disease management. A deeper understanding of these regional differences in immunohematology is critical for tailoring effective treatment strategies and improving outcomes for HIV-infected individuals (Table 1)^[12,21,22]^.

**Table 1:** Comparative Overview of Hematological Markers of HIV in Africa and Russia

Hematological Marker	Africa	Russia
Anemia	Predominantly macrocytic anemia; linked to zidovudine (AZT), malnutrition, chronic infections (e.g., TB)	Mostly normocytic or microcytic; often associated with hepatitis C, ART, or chronic inflammation
Neutropenia	Common in advanced HIV; exacerbated by malnutrition and endemic infections	Linked to ART-induced myelosuppression; less frequent due to earlier ART access
Thrombocytopenia	Early manifestation; often immune-mediated; worsened by co-infections (e.g., malaria)	Related to liver dysfunction (alcohol, hepatitis C), sometimes immune-mediated
CD4 + T Cell Count	Severely reduced at diagnosis due to late presentation and limited screening	Moderately decreased; earlier detection in urban centers leads to higher counts at diagnosis
CD8 + T Cells	Elevated due to chronic immune activation; CD4/CD8 ratio often <0.5	Elevated in early HIV but may normalize with ART; influenced by HCV coinfection
NLR (Neutrophil/Lymphocyte Ratio)	Elevated in advanced disease, TB co-infection; used as inflammatory and prognostic marker	Studied in research contexts; potential correlation with viral load and disease severity
PLR (Platelet/Lymphocyte Ratio)	Investigated in late-stage disease and ART monitoring	Understudied; emerging interest in correlation with inflammation and liver disease
RDW (Red Cell Distribution Width)	Often increased; reflects nutritional deficiencies and chronic inflammation	Increased in HIV/HCV co-infection; under evaluation as a prognostic marker
Coinfection Influence	High burden of TB, malaria, helminthiasis	Hepatitis B/C, tuberculosis, syphilis, STDs

## Aim

The aim of this review is to critically examine and compare the hematological and immunohematologic profiles of people living with HIV in African and Russian populations, with a specific focus on cytopenias and immune dysregulation.

## Review methods

This review employed a structured narrative approach to synthesize current knowledge on hematological and immunohematologic variations among HIV-infected individuals in African and Russian populations. The literature search was conducted using electronic databases such as PubMed, Scopus, Google Scholar, and Web of Science, covering publications from 2000 to 2024. The keywords used included combinations of “HIV,” “hematological markers,” “cytopenia,” “Africa,” “Russia,” “immunohematology,” “ethnoracial differences,” and “antiretroviral therapy.” Boolean operators (AND, OR) were applied to optimize the sensitivity of the search strategy. Peer-reviewed articles, review papers, clinical trials, and government or institutional reports that discussed hematological parameters in HIV-infected individuals within African or Russian settings were included. Studies focusing on both adult and pediatric populations were considered, provided they presented data on hematologic indices such as hemoglobin concentration, white blood cell and platelet counts, CD4+ T-cell levels, and other relevant immune or bone marrow findings. Priority was given to research that drew ethnoracial comparisons or provided insight into regional comorbidities, co-infections, and treatment outcomes. The selected studies were screened for relevance based on titles and abstracts, followed by full-text review to ensure methodological quality and data integrity. Thematic synthesis was used to identify patterns, contrasts, and implications across the included studies. The data were organized under key headings such as hematologic manifestations, regional co-morbidities, diagnostic and therapeutic implications, and clinical outcomes. Special attention was paid to identifying knowledge gaps and inconsistencies in hematologic interpretation between African and Russian populations. By integrating diverse sources and perspectives, this review offers a comprehensive, comparative lens on the intersection of hematology and HIV care in two distinct global regions.

**HIGHLIGHTS**

- Reveals regional disparities in HIV-related hematologic profiles across continents.
- Highlights anemia and neutropenia as consistent global hematologic markers.
- Explores influence of nutrition, co-infections, and ART on blood indices.
- Identifies potential biomarkers for immune monitoring in diverse populations.
- Proposes region-specific hematological thresholds to improve HIV care outcomes.

## Hematological complications of HIV

HIV primarily targets the immune system, but its effects are far-reaching, impacting multiple organ systems, including the hematologic system. HIV-related hematological complications are a critical concern in clinical management as they can exacerbate the morbidity and mortality of the infection. These complications arise due to the direct impact of HIV on the bone marrow, immune dysfunction, and the effects of opportunistic infections and comorbid conditions. As HIV progresses, the virus alters normal hematopoiesis, leading to disorders such as anemia, neutropenia, and thrombocytopenia, each of which plays a significant role in disease progression and patient outcomes^[22,23]^. Anemia, the most common hematological complication in HIV, is observed in up to half of HIV-infected individuals, especially in those with advanced disease or those co-infected with tuberculosis, malaria, or other endemic infections. HIV-related anemia is primarily due to a combination of mechanisms, including bone marrow suppression, hemolysis, and chronic inflammation. The virus directly impacts the bone marrow, reducing its capacity to produce red blood cells. Additionally, HIV-associated chronic inflammation increases the levels of inflammatory cytokines, such as interleukin-6 and tumor necrosis factor-alpha, which interfere with red blood cell production. Co-infections such as tuberculosis, a common comorbidity in HIV-infected individuals, also contribute to anemia through their effects on the bone marrow and by causing increased red blood cell destruction. Nutritional deficiencies, including iron, vitamin B12, and folate, further complicate anemia in HIV-infected patients, especially in resource-limited settings where malnutrition is prevalent^[24–26]^.

Thrombocytopenia, characterized by a low platelet count, is another common hematological abnormality seen in HIV-infected individuals. Platelet dysfunction and a decrease in platelet production are frequently observed in HIV-positive patients, particularly in those with advanced disease or those who have developed opportunistic infections. The pathogenesis of thrombocytopenia in HIV is multifactorial. HIV itself can directly infect megakaryocytes, the precursors of platelets, leading to impaired platelet production in the bone marrow. Additionally, the virus induces immune-mediated platelet destruction, a process further exacerbated by opportunistic infections or malignancies associated with HIV, such as Kaposi’s sarcoma or lymphoma. In some cases, thrombocytopenia may also be exacerbated by liver disease, commonly seen in HIV patients co-infected with hepatitis B or C, as the liver is involved in thrombopoiesis (platelet production)^[27,28]^. Neutropenia, a condition characterized by an abnormally low number of neutrophils (a type of white blood cell), is another hematological complication that is often observed in HIV-infected individuals. Neutropenia increases susceptibility to infections, particularly bacterial and fungal infections, making it a significant contributor to morbidity in these patients. The direct effect of HIV on the bone marrow, as well as the associated immune dysregulation, contributes to the development of neutropenia. In addition to HIV itself, the use of antiretroviral therapy (ART) has been associated with neutropenia in some patients, particularly with drugs like zidovudine, a nucleoside reverse transcriptase inhibitor (NRTI). Moreover, infections such as cytomegalovirus (CMV) or fungal diseases, which are common in HIV-infected individuals, can further reduce neutrophil counts, increasing the risk of life-threatening infections^[29,30]^.

Hematological abnormalities in HIV-infected patients often present challenges in clinical management. The clinical significance of these abnormalities extends beyond just the direct effects on blood cell counts; they are indicative of the underlying immune dysfunction and are often associated with worse disease outcomes. For instance, anemia in HIV patients is associated with higher mortality rates, poor quality of life, and an increased risk of opportunistic infections. The management of hematological complications requires a comprehensive approach, addressing both the HIV infection and any coexisting conditions or comorbidities. In addition to antiretroviral therapy (ART), which can improve overall immune function and reduce the viral load, supportive treatments such as blood transfusions, erythropoiesis-stimulating agents, and platelet transfusions may be necessary to manage severe hematological complications^[31–33]^. Furthermore, the global variation in the presentation of hematological complications in HIV-infected individuals underscores the need for region-specific diagnostic and therapeutic approaches. In resource-limited settings, where co-infections such as tuberculosis and malaria are more prevalent, managing hematological issues requires an integrated approach that also targets these co-infections. On the other hand, in high-income countries, where opportunistic infections may be less common but comorbidities like hepatitis or alcohol use are more prominent, treatment strategies may need to focus more on managing these additional factors. Thus, understanding the unique hematological challenges in HIV care is critical to improving outcomes and providing personalized treatment for individuals across different global regions^[34,35]^.

## Comparative hematological markers in Africa and Russia

The hematological profile of individuals living with HIV provides a window into the complex interplay between viral pathogenesis, host immunity, environmental exposures, and ethnoracial biology. When comparing hematological markers between African and Russian populations living with HIV, distinctive trends emerge, shaped by genetics, comorbidities, healthcare access, and socio-cultural factors. These differences not only reflect biological variability but also influence disease progression, response to treatment, and outcomes. A nuanced understanding of these variations is essential for clinicians and researchers striving to deliver equitable and effective HIV care across different global contexts^[29,36]^. In African populations, the hematological landscape in HIV is often compounded by endemic infections, nutritional deficiencies, and late presentation of disease. Anemia is particularly pervasive, often severe, and multifactorial in origin^[37]^. Malaria, helminthic infections, tuberculosis, and chronic malnutrition frequently co-occur with HIV, each contributing to hematologic depletion through bone marrow suppression, hemolysis, or impaired erythropoiesis. Furthermore, genetic conditions like sickle cell disease or glucose-6-phosphate dehydrogenase (G6PD) deficiency, more prevalent in African populations, can further distort hematological baselines and complicate the interpretation of HIV-related cytopenias. It is not uncommon for African patients to present with pancytopenia by the time of diagnosis, highlighting the compounded burden of systemic inflammation and bone marrow exhaustion^[38]^.

In contrast, HIV-positive populations in Russia exhibit hematological abnormalities that are shaped by a different set of clinical and social determinants. Alcohol-related bone marrow suppression, co-infection with hepatitis C virus (HCV), and injection drug use are prominent contributors to hematologic dysfunction. While anemia and thrombocytopenia are observed in Russian patients, their etiology often leans toward toxic, hepatic, or drug-induced marrow injury rather than infectious or nutritional causes. Russian HIV patients also face challenges associated with late diagnosis, particularly among marginalized populations, which may result in more pronounced immunosuppression and advanced cytopenias at the point of clinical engagement. Importantly, the patterns of antiretroviral therapy (ART) use and adherence in Russia differ from those in many African settings, influencing the trajectory and severity of hematologic changes^[36]^. From an immunohematologic perspective, differences in baseline immune activation and inflammatory profiles also distinguish African and Russian cohorts. Africans may exhibit higher levels of baseline immune activation due to constant antigenic exposure from endemic pathogens, which influences lymphocyte turnover and CD4+ dynamics. Russians, on the other hand, may show immunosuppression linked more to substance use and metabolic dysfunction, particularly among males. These differences manifest in variable white blood cell profiles, CD4:CD8 ratios, and neutrophil-to-lymphocyte ratios, all of which have prognostic implications in HIV care. Additionally, variations in access to diagnostic technologies, laboratory infrastructure, and standardized reference ranges complicate cross-regional comparisons of these markers^[29,37]^. Ultimately, the hematological markers observed in African and Russian HIV populations tell two different but equally critical stories—one shaped by infectious burden and under-resourced health systems, the other by lifestyle-related comorbidities and systemic barriers to care. Bridging the gap between these narratives requires global efforts in research collaboration, culturally tailored diagnostic frameworks, and inclusive clinical guidelines that acknowledge and respond to the realities of diverse HIV-infected populations. Understanding these comparative hematological patterns is not merely an academic exercise—it is fundamental to refining therapeutic strategies, improving prognostic accuracy, and advancing global health equity in the era of HIV^[38]^.

## Diagnostic implications

The diagnostic evaluation of HIV-infected individuals requires a tailored approach that considers the ethnoracial and geographic-specific hematological profiles observed in different populations. In the context of African and Russian populations, hematological markers serve as both indicators of disease severity and reflections of underlying comorbidities and environmental exposures. These differences have significant diagnostic implications, influencing how clinicians interpret laboratory findings, assess disease progression, and initiate timely interventions^[7,11,39]^. In African populations, for instance, baseline hematological values such as hemoglobin levels, white blood cell counts, and platelet indices often differ from Western reference ranges due to genetic factors, endemic infections, and chronic nutritional deficiencies. Consequently, diagnostic thresholds for cytopenias may need to be adjusted to reflect local baselines. For example, anemia in HIV-positive Africans might be overlooked if assessed using generalized thresholds, thereby delaying further evaluation of underlying causes such as tuberculosis, malaria, or bone marrow suppression. Moreover, the high prevalence of co-infections often produces overlapping hematologic abnormalities, complicating the differential diagnosis. This necessitates a broader diagnostic lens that considers both HIV-specific and non-HIV-related contributors to hematological dysregulation^[13,24,25]^. In Russian populations, the diagnostic landscape is shaped more by substance use, alcohol-related liver dysfunction, and co-infection with hepatitis C virus (HCV). These conditions are associated with distinct hematologic manifestations, including macrocytic anemia, leukopenia, and thrombocytopenia stemming from bone marrow suppression or hypersplenism. Thus, in this context, hematological markers not only aid in monitoring HIV progression but also serve as early warning signs of hepatic and hematopoietic toxicity. CD4+ T-cell counts and the CD4/CD8 ratio remain central to immunological assessment; however, these markers may be affected by lifestyle-related immunosuppression, necessitating careful correlation with clinical history. In regions where stigma and healthcare access disparities persist, hematologic screening may offer a critical, non-invasive avenue for initial HIV suspicion and further confirmatory testing^[40–42]^.

## Therapeutic approaches and regional variations

The management of HIV and its hematological complications is profoundly influenced by regional dynamics, ranging from the availability of medical resources to the prevailing co-morbid conditions that shape therapeutic decisions. In comparing African and Russian populations, these regional variations are reflected not only in the clinical presentations of hematologic abnormalities but also in how treatment protocols are designed, accessed, and implemented. A one-size-fits-all approach to therapy is insufficient in such diverse settings, where hematological responses to HIV and its treatment intersect with unique environmental, genetic, and healthcare infrastructure factors. In Africa, the therapeutic approach to HIV-related hematologic complications is often challenged by limited diagnostic infrastructure and constrained drug availability. Anemia, the most prevalent cytopenia among HIV-positive Africans, is frequently managed with iron supplements, nutritional support, and empirical treatment for parasitic or opportunistic infections. However, access to erythropoietin-stimulating agents, blood transfusions, or bone marrow assessments is often restricted to tertiary care centers, leaving many patients untreated or undertreated. Additionally, some antiretroviral drugs, such as zidovudine (AZT), can exacerbate anemia or neutropenia, necessitating careful selection of ART regimens based on baseline hematologic profiles. The implementation of ART guidelines is further complicated by co-infections like tuberculosis and malaria, which may interact pharmacologically or immunologically with HIV therapies and require modifications in treatment schedules and supportive care^[43–45]^.

In contrast, the therapeutic landscape in Russia presents a different set of challenges and opportunities. While more advanced diagnostic and treatment facilities are generally available, socio-behavioral factors such as high rates of intravenous drug use and alcohol dependency significantly influence therapeutic outcomes. These comorbidities can alter the metabolism and effectiveness of ART and hematological treatments, requiring integrated care models that combine addiction services with HIV and hematology care. In Russian settings, thrombocytopenia and leukopenia may be managed with corticosteroids or immunoglobulin therapy when immune-mediated, while alcohol-induced bone marrow suppression necessitates both hematologic and addiction-focused interventions. Furthermore, hepatitis C virus (HCV) co-infection, common among Russian HIV patients, adds another layer of complexity, as antiviral treatment may further impact hematologic parameters and drug tolerability^[46–48]^. Across both regions, the successful management of HIV-related hematologic abnormalities hinges on timely diagnosis, ART regimen optimization, and appropriate supportive care. However, systemic barriers such as healthcare access inequities, stigma, and inconsistent drug supply chains remain substantial hurdles. Tailoring therapy to regional realities—whether by strengthening blood banking systems in sub-Saharan Africa or integrating psychosocial support into HIV care in Russia—is crucial. Importantly, there is a growing recognition of the need for personalized medicine approaches that consider genetic polymorphisms, drug metabolism differences, and ethnoracial hematologic norms to optimize outcomes^[49–51]^.

## Conclusion

The exploration of hematological variations and immunohematologic responses among African and Russian populations living with HIV reveals a complex interplay of genetic, environmental, and socio-economic factors that shape disease manifestation and clinical outcomes. While both populations experience significant hematologic complications such as anemia, leukopenia, and thrombocytopenia, the underlying causes and patterns vary widely due to differences in co-infections, nutritional status, lifestyle factors, and access to healthcare infrastructure. These regional disparities highlight the limitations of a universal clinical approach and emphasize the necessity of context-specific diagnostics and therapies. The comparative analysis underscores the importance of incorporating ethnoracial baselines into hematologic assessments to avoid misdiagnosis and ensure timely, effective treatment. Additionally, regional influences—from endemic diseases in Africa to alcohol-related marrow suppression and hepatitis C co-infection in Russia—demand nuanced clinical management strategies. The findings advocate for the integration of hematologic monitoring into HIV care as a standard practice, especially in resource-limited settings where such indices can serve as early warning systems for disease progression or therapeutic toxicity.

## Data Availability

No datasets were generated or analyzed during the current study.

## Research registration unique identifying number (UIN)

Not applicable as this a narrative review.
